# Clonidine Protects Endothelial Cells from Angiotensin II-Induced Injury via Anti-Inflammatory and Antioxidant Mechanisms

**DOI:** 10.3390/life15081193

**Published:** 2025-07-27

**Authors:** Bekir Sıtkı Said Ulusoy, Mehmet Cudi Tuncer, İlhan Özdemir

**Affiliations:** 1 Department of Interventional Radiology, University of Health Sciences, Gaziantep City Hospital, 27470 Gaziantep, Turkey; bsaidulusoy@hotmail.com; 2 Department of Anatomy, Faculty of Medicine, Dicle University, 21280 Diyarbakir, Turkey; 3 Department of Gynecology and Obstetrics, Faculty of Medicine, Atatürk University, 25030 Erzurum, Turkey; ilhanozdemir25@yandex.com

**Keywords:** cerebral aneurysm, endothelial dysfunction, angiotensin II, clonidine, oxidative stress, inflammation

## Abstract

**Background:** Cerebral aneurysm (CA) is a focal or diffuse pathological dilation of the cerebral arterial wall that arises due to various etiological factors. It represents a serious vascular condition, particularly affecting the elderly, and carries a high risk of rupture and neurological morbidity. Clonidine (CL), an α2-adrenergic receptor agonist, has been reported to suppress aneurysm progression; however, its underlying molecular mechanisms, especially in relation to cerebral endothelial dysfunction, remain unclear. This study aimed to investigate the potential of CL to mitigate CA development by modulating apoptosis, inflammation, and oxidative stress in an Angiotensin II (Ang II)-induced endothelial injury model. **Methods:** Human brain microvascular endothelial cells (HBMECs) were used to establish an in vitro model of endothelial dysfunction by treating cells with 1 µM Ang II for 48 h. CL was administered 2 h prior to Ang II exposure at concentrations of 0.1, 1, and 10 µM. Cell viability was assessed using the MTT assay. Oxidative stress markers, including reactive oxygen species (ROS) and Nitric Oxide (NO), were measured using 2′,7′–dichlorofluorescin diacetate (DCFDA). Gene expression levels of vascular endothelial growth factor (VEGF), matrix metalloproteinases (MMP-2 and MMP-9), high mobility group box 1 (HMGB1), and nuclear factor kappa B (NF-κB) were quantified using RT-qPCR. Levels of proinflammatory cytokines; tumor necrosis factor-alpha (TNF-α), Interleukin-6 (IL-6), and interferon-gamma (IFN-γ); were measured using commercial ELISA kits. **Results:** Ang II significantly increased ROS production and reduced NO levels, accompanied by heightened proinflammatory cytokine release and endothelial dysfunction. MTT assay revealed a marked decrease in cell viability following Ang II treatment (34.18%), whereas CL preserved cell viability in a concentration-dependent manner: 44.24% at 0.1 µM, 66.56% at 1 µM, and 81.74% at 10 µM. CL treatment also significantly attenuated ROS generation and inflammatory cytokine levels (*p* < 0.05). Furthermore, the expression of VEGF, HMGB1, NF-κB, MMP-2, and MMP-9 was significantly downregulated in response to CL. **Conclusions:** CL exerts a protective effect on endothelial cells by reducing oxidative stress and suppressing proinflammatory signaling pathways in Ang II-induced injury. These results support the potential of CL to mitigate endothelial injury in vitro, though further in vivo studies are required to confirm its translational relevance.

## 1. Introduction

Cerebral aneurysms are focal dilatations of the cerebral arterial wall that can lead to serious neurological complications, including subarachnoid hemorrhage and elevated mortality risk upon rupture. The pathogenesis of cerebral aneurysms involves several interrelated mechanisms, including hemodynamic stress, chronic inflammation, vascular smooth muscle cell degeneration, and, most notably, endothelial dysfunction [1,2]. Endothelial cells are essential for maintaining vascular integrity, providing an antithrombotic barrier, and modulating inflammatory responses. Loss of endothelial function compromises the structural stability of the vascular wall, thereby promoting aneurysm development. Among the mechanisms contributing to endothelial dysfunction, apoptosis is a major contributor to endothelial cell loss and weakening of the vessel wall. Apoptosis represents a key cellular response to oxidative and inflammatory stress and has been observed at elevated levels in the walls of ruptured cerebral aneurysms, often accompanied by Extracellular Matrix (ECM) degradation [3]. This pathological remodeling reduces the mechanical strength of the aneurysm wall, increasing the likelihood of rupture.

Ang II is a potent vasoconstrictor known for its role in blood pressure regulation, but it also contributes to vascular pathology through its proinflammatory, pro-oxidative, and pro-apoptotic properties [4]. Ang II stimulates the generation of ROS via NADPH oxidase activation, which initiates mitochondrial apoptotic pathways in endothelial cells [5]. Furthermore, Ang II disrupts endothelial integrity by upregulating proinflammatory cytokines such as IL-6 and TNF-α, as well as adhesion molecules including Vascular Cell Adhesion Molecule-1 and Intercellular Adhesion Molecule-1, thereby promoting vascular inflammation and endothelial barrier breakdown [6].

Although CL is primarily known as a centrally acting α2-adrenergic receptor agonist, it has recently gained attention for its antioxidative and anti-inflammatory effects within the peripheral vascular system. Studies have shown that CL modulates endothelial stress responses by attenuating sympathetic nervous system activity and reducing the production of ROS [7]. Additionally, CL has been reported to suppress apoptotic signaling pathways by maintaining mitochondrial membrane potential, thereby enhancing endothelial cell survival [8]. Despite these promising effects, the potential protective role of CL on cerebral endothelial cells—particularly in the context of Ang II-induced oxidative stress, inflammation, and apoptosis—remains inadequately explored.

This study aimed to evaluate the protective effects of CL against endothelial cell dysfunction and apoptosis induced by Ang II in an in vitro model that mimics early molecular events associated with CA pathogenesis. Specifically, the effects of CL on endothelial cell viability, oxidative stress (ROS and NO levels), proinflammatory cytokine release, and apoptosis-related gene expression were examined. By targeting key pathways involved in vascular inflammation and degeneration, the study sought to explore CL’s potential as a pharmacological agent for mitigating endothelial damage and thereby contributing to aneurysm prevention.

In the present study, we employed an in vitro model of endothelial dysfunction using HBMECs exposed to Ang II to simulate the early pathological processes associated with in vitro endothelial injury model simulating cerebral aneurysm-related pathophysiology formation. Although this cellular model does not fully recapitulate the complex hemodynamic and structural changes seen in true aneurysms, it effectively mimics key features of the endothelial injury, oxidative stress, and proinflammatory signaling that are known contributors to aneurysmal wall degeneration. As such, it provides a controlled and reproducible platform for evaluating pharmacological interventions targeting vascular inflammation and oxidative damage in a context relevant to neurovascular pathology.

## 2. Methods

### 2.1. Cell Culture

Immortalized human brain microvascular endothelial cells (HBMECs; ATCC® CRL-3245™) were obtained from the American Type Culture Collection (ATCC®, Manassas, VA, USA) and used in this study. The cells were cultured in Endothelial Cell Growth Medium (ECGM; PromoCell GmbH, Heidelberg, Germany) supplemented with 10% fetal bovine serum (FBS) and 1% penicillin–streptomycin. Cultures were maintained at 37 °C in a humidified incubator with 5% CO_2_. Once the cells reached approximately 80% confluency, they were subcultured using 0.25% trypsin-EDTA. All experiments were conducted using cells at passages 3 to 5. 

### 2.2. Establishment of an In Vitro Endothelial Injury Model Relevant to Cerebral Aneurysm Pathophysiology and Treatment with CL

**Figure 1:**
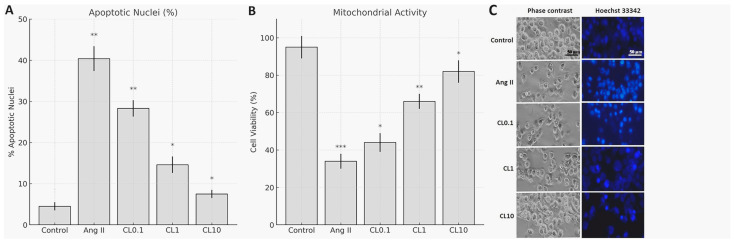
Protective effects of CL on Ang II-induced endothelial injury in HBMECs. (**A**) Quantitative analysis of cell viability assessed by MTT assay. Ang II significantly reduced mitochondrial activity compared to the control group (** *p* < 0.01, *** *p* < 0.001). Pre-treatment with CL at 0.1, 1, and 10 µM restored viability in a concentration-dependent manner (* *p* < 0.05, ** *p* < 0.01, *** *p* < 0.001 vs. Ang II group). (**B**) Quantification of apoptotic nuclei based on Hoechst 33342 staining. Ang II increased the apoptotic index to 40.4% (** *p* < 0.01, *** *p* < 0.001) compared to 4.5% in the control group (* *p* < 0.05). CL significantly reduced apoptosis in a dose-dependent fashion, with 28.3% (** *p* < 0.01), 14.6% (* *p* < 0.05), and 5.0% (* *p* < 0.05) apoptotic nuclei in the CL0.1, CL1, and CL10 groups, respectively. (**C**) Representative phase-contrast and Hoechst 33342-stained fluorescence microscopy images showing nuclear morphological changes. Ang II induced chromatin condensation and nuclear fragmentation, which were progressively alleviated with increasing CL concentrations. Scale bar: 50 µm.

To establish endothelial dysfunction, HBMECs were treated with 1 µM Ang II (Ang II; Sigma-Aldrich, St. Louis, MO, USA) for 24 h. CL (Sigma-Aldrich) was dissolved in sterile distilled water according to the manufacturer’s instructions and diluted to final concentrations of 0.1 µM, 1 µM, and 10 µM. These concentrations were selected based on previous studies demonstrating CL’s endothelial protective effects within this range [7,8], pilot experiments confirming non-toxicity (viability > 90%) and concentration-dependent efficacy in HBMECs (Figure 1), and clinical relevance, as 0.1–10 µM spans subclinical to supraphysiological ranges while avoiding cytotoxicity observed at higher concentrations (>20 µM). CL was administered to the cells 2 h before Ang II exposure. To ensure that observed effects were due to CL itself and not the vehicle, the control group received the same volume of sterile distilled water as the vehicle control. The experimental groups consisted of (i) control (vehicle only), (ii) Ang II only, (iii) Ang II + CL 0.1 µM (CL0.1), (iv) Ang II + CL 1 µM (CL1), and (v) Ang II + CL 10 µM (CL10).

Although plasma concentrations of Ang II in vivo are typically in the picomolar to nanomolar range, the concentration of 1 µM was selected based on prior studies in endothelial cell cultures, where it consistently induces robust oxidative and inflammatory responses [4,5]. This relatively high dose accounts for the lack of in vivo clearance mechanisms and mimics localized vascular Ang II accumulation observed in pathophysiological states such as cerebral aneurysms or hypertension [9].

### 2.3. Cell Viability Test

Cells were seeded into 96-well plates at a density of 1 × 10^3^ cells per well. Following seeding, the plates were incubated in a humidified CO_2_ incubator at 37 °C for 24 h to allow for cell attachment. After incubation, cells were treated with 1 µM Ang II alone or in combination with CL at concentrations of Ang II + CL 0.1 µM (CL0.1), Ang II + CL 1 µM (CL1), and Ang II + CL 10 µM (CL10) for an additional 24 h. Following treatment, the culture medium was removed, and the MTT assay was performed as described by Mosmann [10]. Briefly, 20 µL of 3-(4,5-dimethylthiazol-2-yl)-2,5-diphenyltetrazolium bromide (MTT; Sigma-Aldrich) solution was added to each well, and the volume was adjusted to 100 µL with ECGM. Plates were incubated for 2 h at 37 °C in the dark. After incubation, absorbance was measured at 490 nm using a microplate reader, with a reference wavelength of 630 nm. For apoptotic nuclear morphology assessment, cells were stained with Hoechst 33342 (5 μg/mL) for 10 min at 37 °C. Apoptotic cells (condensed/fragmented nuclei) were quantified under fluorescence microscopy (20× magnification). Three random fields per well were analyzed, and the apoptotic index was calculated as (apoptotic nuclei/total nuclei) × 100.

### 2.4. Determination of ROS and NO Levels

#### 2.4.1. Determination of ROS Levels

Intracellular ROS levels were measured using DCFDA Cellular ROS Detection Assay Kit (Sigma Aldrich, Cat: 287810), following the manufacturer’s protocol. After treatment with Ang II and CL, cells were incubated with 25 µM DCFDA at 37 °C in the dark for 45 min. Subsequently, the cells were washed with phosphate-buffered saline (PBS), and fluorescence intensity was measured at 485 nm excitation and 535 nm emission wavelengths using a fluorescence plate reader (BioTek, Synergy H1, BioTek Instruments, Inc., Winooski, VT, USA). ROS levels were normalized to protein content and expressed as relative fluorescence units per mg protein (RFU/mg).

#### 2.4.2. Determination of NO Levels

NO production was determined indirectly by measuring nitrite accumulation using the Griess Reagent Assay (Thermo Scientific, Waltham, MA, USA) [11]. After treatment, 50 µL of cell culture supernatant was mixed with an equal volume of Griess reagent (1% sulfanilamide, 0.1% N-(1-naphthyl)ethylenediamine dihydrochloride in 2.5% phosphoric acid) in a 96-well plate and incubated at room temperature for 10 min in the dark. Absorbance was read at 540 nm using a microplate reader (BioTek). Nitrite concentrations were calculated using a sodium nitrite standard curve and expressed as µM/mg protein.

### 2.5. Quantification of Proinflammatory Cytokines by ELISA

Levels of the inflammatory cytokines TNF-α, IL-6, and IFN-γ were quantified in HBMEC lysates using commercially available sandwich ELISA kits (Invitrogen, Carlsbad, CA, USA; Cat. #13-7341-81 for TNF-α, #EH2IL6 for IL-6, and #14-7311-81 for IFN-γ), following the manufacturer’s protocols. Briefly, 96-well plates pre-coated with specific capture antibodies were incubated with prepared cell lysates. After washing, wells were incubated with biotin-conjugated detection antibodies, followed by streptavidin-HRP. The enzymatic reaction was developed using a chromogenic substrate, and absorbance was measured at 540 nm using a microplate reader. Cytokine concentrations were normalized to the total protein content of each sample and expressed as ng/mg or pg/mg of protein.

### 2.6. Gene Expression Analysis (RT-qPCR)

Total RNA was extracted from treated HBMECs using TRIzol Reagent (Invitrogen), and complementary DNA (cDNA) was synthesized using the RevertAid First Strand cDNA Synthesis Kit (Thermo Scientific), following the manufacturer’s protocols. Quantitative real-time PCR (RT-qPCR) was performed using the QuantStudio 3 Real-Time PCR System (Applied Biosystems, Foster City, CA, USA) with SYBR Green Master Mix. The expression levels of VEGF, MMP-2, MMP-9, HMGB1, and NF-κB p65 were assessed using specific primer sequences listed in Table 1. GAPDH was used as the internal reference gene. Relative gene expression was calculated using the 2^−ΔΔCt^ method. Primer efficiency was calculated using standard curves generated from serial cDNA dilutions, and all primer sets showed efficiencies between 90 and 110%. Additionally, melt curve analysis was performed for each qPCR reaction and yielded a single distinct peak, confirming specific amplification and excluding nonspecific products or primer-dimer artifacts.

### 2.7. Bioinformatics/GO Analysis

To elucidate the functional relevance of MMP-2 and MMP-9 in the context of cerebral endothelial dysfunction, Gene Ontology (GO) enrichment analysis was performed. The analysis categorized gene functions into three principal domains: Biological Process (BP), Molecular Function (MF), and Cellular Component (CC). MMP-2 and MMP-9 were primarily associated with ECM remodeling, inflammatory signaling, and angiogenesis. Notably, MMP-driven inflammation was linked to upregulation of NF-κB, a central transcription factor in vascular inflammation, while pathological angiogenesis was mediated through VEGF signaling.

GO enrichment analysis was conducted to investigate the biological functions, molecular activities, and subcellular localizations of the genes associated with endothelial dysfunction. The analysis was performed using STRING and ShinyGO (v0.76), and results were classified into three main GO categories: BP, MF, and CC.

### 2.8. Statistical Analysis

All experiments were conducted with a minimum of three independent biological replicates. Data are expressed as mean ± standard deviation (SD). Statistical comparisons between multiple groups were performed using one-way analysis of variance (ANOVA), followed by Tukey’s post hoc test for pairwise comparisons. Prior to ANOVA, assumptions of normality and homogeneity of variances were evaluated using the Shapiro–Wilk and Levene’s tests, respectively. Differences were considered statistically significant at *p* < 0.05. All analyses were performed using SPSS software version 20.0 (IBM Corp., Armonk, NY, USA).

## 3. Results

### 3.1. Effect of CL on Cell Viability

In the in vitro CA model induced by Ang II, the potential proliferative and protective effects of CL on HBMECs were evaluated. Cell viability was assessed using the MTT assay in accordance with the manufacturer’s protocol. As shown in Figure 1, Ang II treatment resulted in a significant decrease in mitochondrial activity and overall cell viability compared to the control group (*p* < 0.001), confirming the successful induction of endothelial dysfunction. Pre-treatment with CL at concentrations of 0.1, 1, and 10 µM led to a concentration-dependent recovery in cell viability, with viability increasing to 44.24% at 0.1 µM (*p* < 0.05), 66.56% at 1 µM (*p* < 0.01), and 81.74% at 10 µM (*p* < 0.001), respectively.

To visualize the morphological effects of Ang II and CL, HBMECs were stained with Hoechst 33342 and analyzed under fluorescence microscopy (Figure 1). Ang II exposure induced hallmark features of apoptosis, including chromatin condensation and nuclear fragmentation. In contrast, CL treatment ameliorated these nuclear alterations in a concentration-dependent manner, consistent with the viability data.

Quantitative analysis of Hoechst 33342-stained nuclei revealed a marked increase in apoptotic nuclear morphology in the Ang II group, with the apoptotic index rising from 4.5% in the control group to 40.4%. CL treatment significantly reduced the percentage of apoptotic nuclei in a concentration-dependent manner: 28.3% at Ang II + CL 0.1 µM (CL0.1), 14.6% at Ang II + CL 1 µM (CL1), and 7.5% at Ang II + CL 10 µM (CL10), as shown in Figure 1.

Bar graph showing the percentage of apoptotic nuclei in HBMECs following Ang II and CL treatment. Apoptotic cells were identified based on nuclear morphology (condensation, fragmentation, and hyperintensity) observed under Hoechst 33342 staining. Ang II treatment markedly increased apoptotic nuclei (~40.4%) compared to the control group (~4.5%). CL reduced apoptosis in a concentration-dependent manner: 28.3% Ang II + CL 0.1 µM (CL0.1), 14.6% Ang II + CL 1 µM (CL1), and 7.5% Ang II + CL 10 µM (CL10). Data are expressed as percentage of apoptotic nuclei per total counted nuclei (Figure 1).

### 3.2. Effect of CL on ROS and NO Levels in In Vitro Endothelial Injury Model Simulating Cerebral Aneurysm-Related Pathophysiology

**Figure 2:**
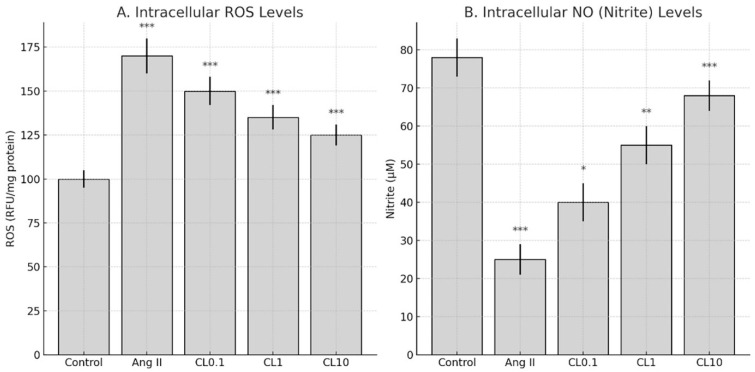
CL attenuates oxidative stress and restores NO levels in HBMECs exposed to Ang II. (**A**) Intracellular ROS levels measured using the DCFDA Cellular ROS Detection Assay. Ang II significantly increased ROS production compared to the control group (*** *p* < 0.001), indicating enhanced oxidative stress. Co-treatment with CL at 0.1, 1, and 10 µM (CL0.1, CL1, CL10) reduced ROS levels in a concentration-dependent manner (*** *p* < 0.001 vs. Ang II group). (**B**) NO levels quantified using the Griess reagent assay. Ang II markedly decreased NO production compared to the control group (*** *p* < 0.001). CL treatment restored NO levels in a dose-dependent manner, with significant increases at CL0.1 (* *p* < 0.05), CL1 (** *p* < 0.01), and CL10 (*** *p* < 0.001) versus the Ang II group. Data are presented as mean ± SD (n = 3). Statistical significance was determined using one-way ANOVA followed by Tukey’s post hoc test.

Intracellular ROS levels were measured using the DCFDA Cellular ROS Detection Kit (Sigma-Aldrich), following the manufacturer’s instructions. HBMECs were treated with CL at concentrations of Ang II + CL 0.1 µM (CL0.1), Ang II + CL 1 µM (CL1), and Ang II + CL 10 µM (CL10) for 24 h following Ang II exposure. Ang II treatment significantly increased intracellular ROS levels compared to the control group (*p* < 0.001), indicating the induction of oxidative stress and endothelial dysfunction, which are critical factors in CA pathogenesis (Figure 2).

CL treatment significantly reduced ROS levels in a concentration-dependent manner. Specifically, 0.1 µM CL caused a modest but statistically significant reduction in ROS intensity (*p* < 0.001 vs. Ang II group), 1 µM CL markedly decreased ROS accumulation (*p* < 0.001), and 10 µM CL restored ROS levels to near-baseline values (*p* < 0.001 vs. Ang II group) (Figure 2).

Ang II treatment resulted in a significant reduction in NO levels in HBMECs compared to the control group (*p* < 0.001). This reduction is a hallmark of endothelial dysfunction and is associated with impaired vascular tone and increased susceptibility to aneurysm formation. CL administration restored NO levels in a concentration-dependent manner. Notably, treatment with Ang II + CL 10 µM led to a statistically significant increase in NO levels compared to the Ang II group (*p* < 0.001), indicating a potential protective effect on endothelial function (Figure 2).

### 3.3. Proinflammatory Cytokine Levels

**Figure 3:**
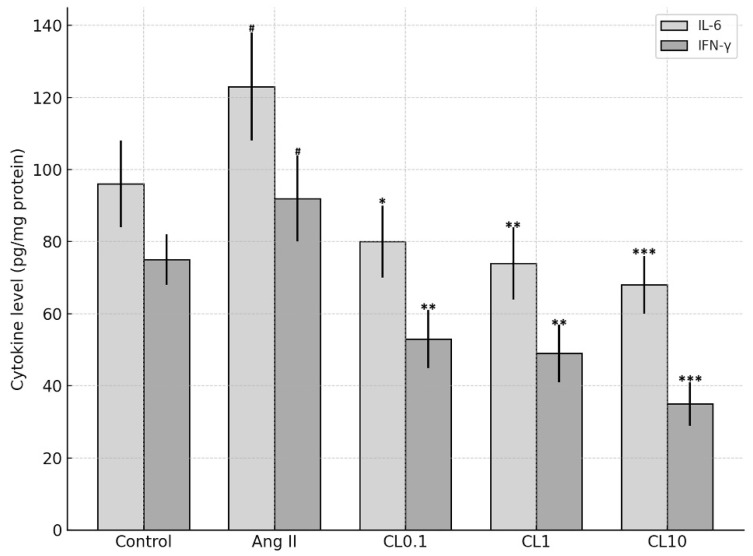
Quantification of IL-6, IFN-γ, and TNF-α levels in HBMECs following Ang II and CL treatment. Bar graph shows the concentrations (pg/mg protein) of IL-6, IFN-γ, and TNF-α in the Control, Ang II, and Ang II + CL (CL0.1, CL1, CL10) groups. Ang II significantly increased the levels of the cytokines compared to the control group (# *p* < 0.001). Co-treatment with CL resulted in a concentration-dependent reduction in cytokine levels, with the most pronounced suppression observed at 10 µM. Data are presented as mean ± SD. Statistical significance vs. the Ang II group is indicated by * *p* < 0.05, ** *p* < 0.01, and *** *p* < 0.001.

**Figure 4:**
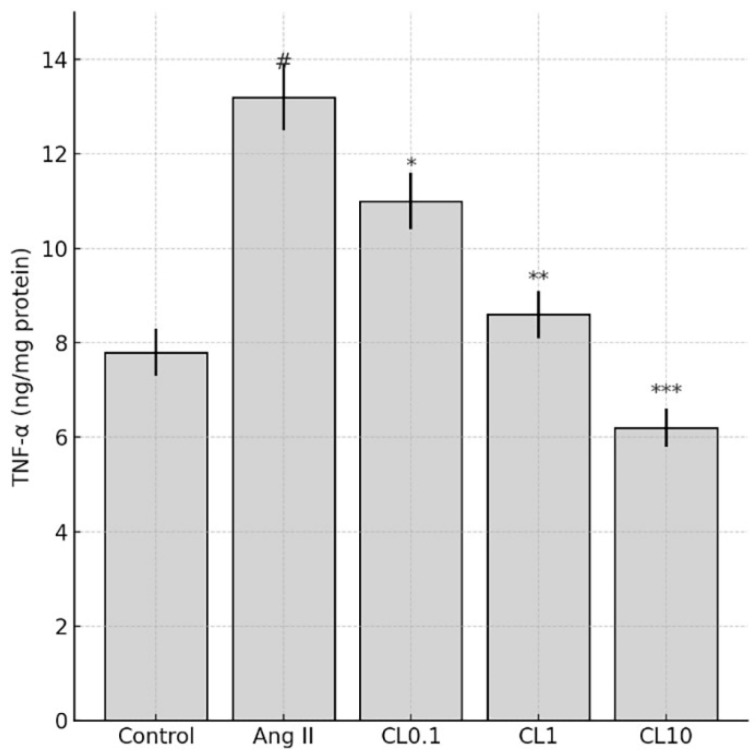
Effects of CL treatment on TNF-α levels in an Ang II-induced CA model. Bar graph illustrates TNF-α levels (ng/mg protein) in HBMECs across five experimental groups: Control, Ang II, and Ang II + CL (CL0.1, CL1, CL10). Ang II administration significantly increased TNF-α levels compared to the control group (# *p* < 0.001). CL treatment reduced TNF-α levels in a concentration-dependent manner, with significant decreases at CL0.1 (* *p* < 0.05), CL1 (** *p* < 0.01), and CL10 (*** *p* < 0.001) versus the Ang II group. Data are presented as mean ± SD.

In our study, the levels of proinflammatory cytokines including TNF-α, IL-6, and IFN-γ were significantly elevated in groups subjected to the Ang II-induced CA model. However, a notable reduction in these cytokines was observed following CL treatment. TNF-α levels decreased by approximately 40% after CL administration (*p* < 0.05), contributing to the suppression of inflammation and the mitigation of tissue damage (Figure 3). IL-6 levels were reduced by approximately 35–45% following treatment (*p* < 0.01), indicating attenuation of the acute inflammatory response. Similarly, IFN-γ levels decreased by 30–40% with CL administration (*p* < 0.01), highlighting its inhibitory effect on Th1-mediated immune activation (Figure 3). These anti-inflammatory effects are further supported by the significant downregulation of TNF-α levels across CL-treated groups, as shown in Figure 4.

### 3.4. RT-qPCR Findings

**Figure 5:**
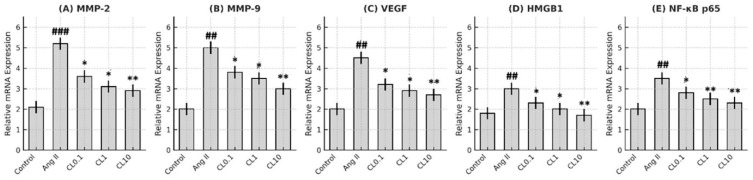
CL attenuates Ang II-induced upregulation of inflammatory and matrix remodeling gene expression in HBMECs. Quantitative real-time PCR analysis of relative mRNA expression levels of (A) MMP-2, (B) MMP-9, (C) VEGF, (D) HMGB1, and (E) NF-κB p65 in Control, Ang II, and Ang II + CL (CL0.1, CL1, CL10) treatment groups. Ang II significantly upregulated the expression of all genes compared to the Control group (## *p* < 0.01, ### *p* < 0.001 vs. Control). CL treatment resulted in a dose-dependent reduction in gene expression levels compared to the Ang II group (* *p* < 0.05, ** *p* < 0.01 vs. Ang II). Data are presented as mean ± SD (n = 3). Statistical comparisons were performed using one-way ANOVA followed by Tukey’s post hoc test.

In our study, the effects of CL treatment on the expression levels of VEGF, MMP-2, MMP-9, HMGB1, and NF-κB p65 genes were evaluated in an in vitro model of CA. A significant reduction in VEGF mRNA expression was observed in all CL-treated groups compared to the Ang II group (*p* < 0.05 for Ang II + CL 0.1 µM (CL0.1), *p* < 0.01 for Ang II + CL 1 µM (CL1), and *p* < 0.001 for Ang II + CL 10 µM (CL10)). This suggests that CL may inhibit the production of VEGFs, thereby reducing pathological vascular remodeling associated with aneurysm formation. Notably, Ang II + CL 10 µM (CL10) treatment resulted in a 44% decrease in MMP-2 expression and a 38% decrease in MMP-9 expression (*p* < 0.001), indicating CL’s potential to mitigate ECM degradation induced by Ang II. In addition, HMGB1 expression was reduced by approximately 50% in the Ang II + CL 10 µM (CL10) group (*p* < 0.001), supporting CL’s role in regulating inflammatory signaling. NF-κB p65 gene expression was also significantly suppressed in CL-treated cells (*p* < 0.01), suggesting that CL may exert its anti-inflammatory effects by downregulating central transcription factors involved in aneurysm pathogenesis (Figure 5).

### 3.5. Gene Ontology Findings

**Figure 6:**
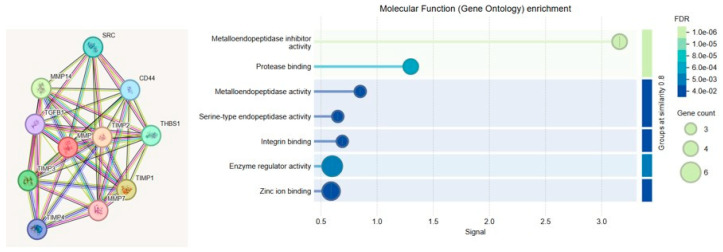
Molecular function enrichment and Protein–Protein Interaction (PPI) network analysis of MMP-related genes. (**Left**) STRING-based PPI network showing the interaction landscape among MMP-2 and MMP-9 and related regulatory proteins, including TIMPs, CD44, THBS1, TGF-β1, and SRC. Nodes represent proteins, and edges indicate predicted functional associations based on curated databases, co-expression, and experimental data. (**Right**) GO enrichment analysis for MF terms, visualized by dot plot. Significantly enriched terms include metalloendopeptidase inhibitor activity, protease binding, and metallopeptidase activity, suggesting the central role of MMP-2 and MMP-9 in enzymatic regulation, proteolysis, and zinc-dependent catalysis. Dot size reflects gene count; color indicates false discovery rate (FDR).

MMP-2 and MMP-9 genes belong to the matrix metalloproteinase family, which plays critical roles in ECM degradation, tissue remodeling, and various pathological processes. In this study, GO analysis was conducted to explore the biological functions, subcellular localizations, and disease associations of MMP-2 and MMP-9, particularly in the context of CA. These enzymes are directly involved in ECM degradation (GO:0030574) and tissue remodeling (GO:0048771). They also contribute significantly to tumor invasion and metastasis (GO:0007162) through their roles in angiogenesis (GO:0001525) and cell migration (GO:0016477). Additionally, MMP-2 and MMP-9 are linked to inflammatory responses (GO:0006954) and are highly expressed in chronic inflammatory conditions such as rheumatoid arthritis and atherosclerosis. These associations are visualized in Figure 6.

A list of individual genes significantly associated with enriched molecular functions is provided in Table 2. Notably, TIMPs and MMPs were involved in metallopeptidase activity, enzyme regulation, and zinc ion binding functions. 

## 4. Discussion

This study demonstrates that CL confers significant protection against Ang II-induced endothelial dysfunction in an in vitro CA model, through its anti-inflammatory and antioxidant properties. Exposure to Ang II markedly reduced HBMEC viability and induced pronounced oxidative stress, as evidenced by increased intracellular ROS and decreased NO levels. These changes were accompanied by elevated expression of proinflammatory cytokines (TNF-α, IL-6, IFN-γ) and genes involved in vascular remodeling and inflammation (MMP-2, MMP-9, VEGF, HMGB1, NF-κB p65). Importantly, CL pre-treatment reversed these pathological alterations in a concentration-dependent manner. Nitric oxide plays a pivotal role in maintaining endothelial integrity by promoting vasodilation, preventing platelet aggregation, and reducing leukocyte adhesion to the vascular wall [12]. In addition, NO exhibits strong antioxidant and anti-apoptotic properties, making it a key regulator of endothelial cell survival under conditions of oxidative and inflammatory stress [13]. In the present study, NO levels were significantly reduced following Ang II exposure, reflecting the impairment of endothelial function. CL treatment restored NO concentrations in a dose-dependent manner, suggesting that part of its protective effect involves the preservation of NO bioavailability. Although we did not directly evaluate the expression of endothelial NO synthase (eNOS), which is the primary enzyme responsible for NO synthesis in endothelial cells, the observed increase in NO levels may indirectly reflect eNOS activity. These findings support the conclusion that CL contributes to endothelial protection not only by attenuating oxidative and inflammatory damage but also by enhancing NO-mediated signaling pathways.

The observed increase in cell viability, reduction in apoptosis, normalization of NO/ROS balance, and downregulation of inflammatory and remodeling-associated genes suggest that CL can mitigate endothelial injury through multifaceted mechanisms. These findings support the hypothesis that CL targets core pathological features of aneurysm development—including endothelial cell loss, ECM degradation, and chronic inflammation—thereby providing a potential pharmacological strategy for preventing aneurysmal progression. Our findings are consistent with previous reports indicating that Ang II induces oxidative stress and elevates proinflammatory cytokine production in both endothelial and vascular smooth muscle cells. For example, Dandona et al. demonstrated that Ang II suppresses endothelial NO synthase (eNOS) activity and enhances IL-6 and TNF-α production via ROS-dependent pathways in human vascular cells [14]. Benicky et al. further showed that Ang II upregulates TNF-α and IL-6 expression both centrally and peripherally, contributing to vascular inflammation [15]. In our model, treatment with 1 µM Ang II decreased NO levels by approximately 50% and increased TNF-α, IL-6, and IFN-γ levels by 2–3 fold, which falls within the same range as these established studies. Thus, our in vitro model reliably recapitulates key inflammatory and oxidative effects of Ang II, reinforcing its relevance for studying endothelial dysfunction in vitro endothelial injury model simulating cerebral aneurysm-related pathophysiology pathogenesis.

Ang II is a vasoactive peptide widely recognized as a key initiator of vascular inflammation and a critical contributor to CA development. Its pathogenic role is primarily mediated through the generation of ROS, endothelial dysfunction, elevated proinflammatory cytokine production, and transcriptional regulation of vascular remodeling genes [9,16]. Consistent with these findings, our study demonstrated that Ang II treatment led to a marked decrease in cell viability and a significant upregulation of inflammatory mediators, including ROS, IL-6, TNF-α, and IFN-γ. CL, an α2-adrenergic receptor agonist, is traditionally known for its central sympatholytic effects but has also been shown to exert potent antioxidant and anti-inflammatory actions in peripheral vascular systems [17]. Our data support this dual action, showing that CL significantly ameliorated Ang II-induced endothelial dysfunction in a concentration-dependent manner. Notably, treatment with 10 μM CL restored cell viability to near-control levels and substantially reduced both ROS and cytokine levels. Moreover, Ang II-induced overexpression of critical genes involved in aneurysm pathogenesis—such as VEGF, MMP-2, MMP-9, HMGB1, and NF-κB p65—was significantly downregulated following CL treatment. The suppression of VEGF and matrix metalloproteinases is particularly relevant, as these factors contribute to ECM degradation and vascular wall destabilization [18,19]. Additionally, the downregulation of NF-κB p65 indicates that CL may inhibit a central transcriptional regulator of inflammation [20]. Previous studies have also suggested that CL may reduce cAMP levels via a G-protein-coupled mechanism, thereby attenuating MMP expression in Vascular Smooth Muscle Cells (VSMCs) [21]. This, in conjunction with reduced sympathetic outflow and improved vascular tone, may collectively decrease hemodynamic stress—a known contributor to aneurysm progression [22].

CL has been reported to exert diverse physiological effects through multiple mechanisms of action. While some studies have suggested that CL may exacerbate endothelial dysfunction under certain conditions [23], the current study demonstrated that low- concentration Ang II + CL 0.1 µM (CL0.1) was both safe and protective in the context of cerebral endothelial injury. Reactive oxygen species, commonly referred to as free radicals, are typically regulated by endogenous antioxidant defense systems, including superoxide dismutase (SOD), catalase, and glutathione peroxidase. However, an imbalance between ROS production and antioxidant capacity leads to oxidative stress. Mitochondria are the primary intracellular source of ROS, and excessive ROS accumulation can result in damage at the cellular, tissue, and organ levels. Moreover, ROS can inhibit glutamate reuptake and activate NMDA (N-methyl-D-aspartate) receptors, triggering additional superoxide generation, calcium influx, and NO production. CL is also believed to possess antioxidant properties, which may contribute to its protective role in vascular pathologies by attenuating oxidative stress and preserving endothelial function [24]. Previous studies have demonstrated CL’s antioxidant capacity per se. In particular, Gourdin et al. showed that CL significantly reduced oxidative burst and improved endothelial-dependent vasodilation following ischemia–reperfusion injury in humans, supporting its direct role in modulating oxidative stress [7].

Histological evaluations of cerebral aneurysms have consistently demonstrated features of both acute and chronic inflammation, as well as medial wall degeneration [25]. Numerous immunological factors have been implicated in the formation and progression of cerebral aneurysms, along with the underlying mechanisms driving these processes. Excessive hemodynamic stress disrupts endothelial cell function and promotes the activation of inflammatory cells within the vascular media [26]. Key molecular mediators such as NF-κB and Ang II further amplify these responses, contributing to phenotypic changes in VSMCs, which acquire proinflammatory and matrix-remodeling properties [27]. This phenotypic switch initiates apoptotic pathways and facilitates ECM degradation. Matrix metalloproteinases, particularly those secreted by macrophages and VSMCs, play a central role in ECM breakdown and pathological vascular remodeling. Additionally, cytokines such as IL-6, TNF-α, and NO are key regulators of vascular inflammation and immune responses [28]. Elevated levels of these cytokines and associated chemokines have been detected in the bloodstream of patients with cerebral aneurysms [29]. The present study’s findings are notable in that CL treatment led to significant reductions not only in MMP expression but also in TNF-α and IL-6 levels, supporting its dual role in modulating both immune activation and the inflammatory cascade involved in aneurysm pathophysiology.

These results are consistent with previous studies that have demonstrated the protective effects of CL in models of systemic inflammation and vascular injury [30]. However, it is important to acknowledge that the present study was conducted using an in vitro model of cerebral endothelial dysfunction. Therefore, further in vivo investigations using relevant animal models are necessary to validate these findings and to comprehensively evaluate the translational potential of CL as a therapeutic agent for CA prevention. CL, an alpha-2 adrenergic agonist, has demonstrated potential in modulating endothelial function and inflammation. In a study by Taguchi et al., co-treatment with CL and a GRK2 inhibitor in diabetic mice prevented rebound hypertension and endothelial dysfunction after withdrawal. This effect was associated with increased Akt/eNOS signaling and NO production, suggesting a protective role of CL in vascular health [31].

Ang II is known to exert proinflammatory effects on the vascular system. It increases the expression of adhesion molecules, cytokines, and chemokines, leading to endothelial dysfunction. Ang II also promotes oxidative stress by generating ROS, further contributing to vascular inflammation and damage [14,15,32,33,34]. Oxidative stress plays a significant role in the pathogenesis of cerebral aneurysms. It induces endothelial injury and smooth muscle cell phenotypic switching, leading to vessel wall remodeling and potential rupture. Laaksamo et al. reported that oxidative stress is associated with cell death and wall degradation in intracranial aneurysms [35], highlighting its importance in aneurysm progression [36,37,38]. Furthermore, recent studies have identified oxidative stress-related biomarkers in intracranial aneurysms. Zhang et al. utilized machine learning algorithms to identify four key genes associated with oxidative stress in intracranial aneurysms, providing insights into potential diagnostic markers and therapeutic targets [39]. These findings collectively suggest that CL may attenuate Ang II-induced endothelial dysfunction through anti-inflammatory and antioxidant pathways, offering a potential therapeutic approach in conditions like CA pathogenesis.

Despite the robust molecular and cellular findings presented in this study, several limitations should be acknowledged. In addition, while the current study focused on CL’s protective role under Ang II-induced injury, a CL-only group (without Ang II) was not included. This limits the ability to determine whether the observed effects are specific to stress conditions or also occur under baseline physiology. Future studies should include such groups to distinguish between baseline pharmacological activity and stimulus-specific modulation. First, the experimental model employed was based entirely on in vitro assays using HBMECs to simulate cerebral endothelial dysfunction. While this model effectively mimics early-stage endothelial injury and allows for precise mechanistic exploration, it does not fully capture the complex hemodynamic, immunological, and neurovascular interactions present in in vivo CA development. Second, the effects of CL were evaluated over a relatively short treatment period (24 h), and longer-term outcomes such as endothelial repair, matrix stabilization, or gene expression persistence were not assessed. Additionally, while three concentrations of CL were tested, pharmacokinetic data and physiological relevance to systemic administration in animal or clinical settings were not explored. Interestingly, TNF-α levels in the CL10 group were observed to be slightly lower than those in the control group. This phenomenon may be attributed to the strong anti-inflammatory potential of high-dose CL, which has been shown to suppress not only stimulus-induced cytokine production but also baseline (unstimulated) expression levels via inhibition of NF-κB and MAPK pathways. CL-mediated downregulation of intracellular cAMP has also been linked to reduced activation of transcriptional regulators involved in basal cytokine expression. These mechanisms are particularly evident at supraphysiological concentrations, such as 10 µM, which may explain the slight reduction in TNF-α below control levels observed in our study [8]. The anti-inflammatory and antioxidant effects observed—though statistically significant—were measured at the transcriptional and biochemical levels, and thus functional outcomes such as endothelial barrier restoration, vascular tone modulation, or aneurysmal wall stabilization were not directly evaluated.

One limitation of our study is the use of a pretreatment protocol with CL, which may not fully represent the clinical context where treatment is typically initiated after pathology onset. Future in vitro and in vivo studies should include posttreatment protocols to evaluate the therapeutic efficacy of CL under more physiologically relevant conditions. Additionally, future studies should confirm the presence of alpha two adrenergic receptors in HBMECs under our experimental conditions and assess whether CL’s protective effects can be reversed by specific alpha two AR antagonists such as yohimbine. These experiments would provide mechanistic insight into receptor specific actions of CL. Future studies should incorporate in vivo aneurysm models such as elastase induced or genetically modified rodent models to validate the protective effects of CL under physiological and pathological conditions. These models would also allow assessment of aneurysm incidence, rupture rate, and histopathological wall integrity following treatment. Moreover, integration of hemodynamic modeling and imaging modalities such as MRI angiography and Doppler flowmetry could provide valuable insights into the vascular remodeling process in response to CL. Finally, it would be important to examine CL’s interaction with other vasoactive or anti-inflammatory agents as well as its long term safety and efficacy profile in the context of aneurysm prevention. Although gene expression analyses were performed to assess key apoptotic and oxidative stress markers, protein level confirmation such as Western blotting for Caspase 3 or related proteins could not be included due to laboratory constraints. This limitation has now been explicitly acknowledged. We agree that future studies should incorporate immunoblotting or immunohistochemical methods to validate mRNA findings and deepen the mechanistic understanding of CL’s protective effects. Elucidating downstream signaling pathways such as MAPK, PI3K Akt, or JAK STAT and confirming protein level expression changes via immunoblotting or immunohistochemistry will also enhance the mechanistic clarity of its therapeutic action.

## 5. Conclusions

This study provides compelling experimental evidence that CL exerts significant protective effects against Ang II-induced endothelial dysfunction, a key pathological trigger in the development of in vitro endothelial injury model simulating cerebral aneurysm-related pathophysiologys. By restoring endothelial cell viability, reducing apoptotic nuclear changes, and attenuating oxidative and inflammatory stressors—including ROS, NO imbalance, and elevated proinflammatory cytokines—CL demonstrated multifaceted cytoprotective properties in HBMECs. Importantly, the downregulation of angiogenic and matrix-degrading genes such as VEGF, MMP-2, MMP-9, HMGB1, and NF-κB p65 further supports its role in mitigating ECM destabilization and inflammatory amplification—hallmark events in aneurysm pathogenesis. While these findings demonstrate notable cytoprotective effects of CL in an in vitro model, they should be interpreted cautiously within the context of experimental limitations. Further in vivo validation is required to determine whether these protective mechanisms are maintained under physiological and pathological conditions. Based on the present results, CL may warrant further investigation for its potential endothelial-protective properties in neurovascular disease research.

## Figures and Tables

**Table 1:** Primer sequences for Real-time RT-PCR.

GENES	PRıMER SEQUENCES (5′-3′)
*MMP-2*	Forward: 5′-TTGATGGCATCGCTCAGATCC-3′ Reverse: 5′-GGCAGCAGTGGATGTTCTTGG-3′
*MMP-9*	Forward: 5′-AGATCTTCTTCTTCAAGGACCGGTT -3′ Reverse: 5′-GGCTCCTCAGTGGCTTGGGGTA-3′
*NF-κB P65*	Forward: 5′-TTAGCCATCATCCACCTTC-3′ Reverse: 5′-AGTCCTCCACCACATCTT-3′
*VEGF*	Forward: 5′-AGGGCAGAATCATCACGAAGT-3′ Reverse: 5′-AGGGTCTCGATTGGATGGCA-3
*HMGB1*	Forward: 5′-ATATGGCAAAAGCGGACAAG-3′ Reverse: 5′-GCAACATCACCAATG-GACAG-3′

**Table 2:** GO molecular function enrichment.

GO Term (MF)	Enriched Genes	FDR	Gene Count
Metalloendopeptidase inhibitor activity	TIMP1, TIMP2, TIMP3	1.0 × 10^−6^	3
Protease binding	MMP2, MMP9, TIMP1, TIMP2, THBS1, TGFβ1	8.0 × 10^−6^	6
Metalloendopeptidase activity	MMP2, MMP9, MMP14, TGFβ1	1.0 × 10^−5^	4
Serine-type endopeptidase activity	MMP9, THBS1	5.0 × 10^−4^	2
Integrin binding	THBS1, CD44	5.0 × 10^−3^	2
Enzyme regulator activity	TIMP1, TIMP2, TIMP3	8.0 × 10^−3^	3
Zinc ion binding	MMP2, MMP9, MMP14	4.0 × 10^−2^	3

## Data Availability

All details about the study can be obtained from the corresponding author.

## Abbreviations

The following abbreviations are used in this manuscript:

Angiotensin II	Ang II
Biological Process	BP
Cerebral aneurysm	CA
Cellular Component	CC
Clonidine	CL
DCFDA	2′,7′–dichlorofluorescin diacetate
ECM	Extracellular Matrix
Gene Ontology	GO
HBMECs	Human brain microvascular endothelial cells
IFN-γ	Interferon-gamma
IL-6	Interleukin-6
MF	Molecular Function
MMP-2 and MMP-9	Matrix metalloproteinases
NF-κB	Nuclear Factor kappa B
NO	Nitric oxide
PPI	Protein–Protein Interaction
ROS	Reactive oxygen species
TNF-α	Tumor Necrosis Factor-alpha
VEGF	Vascular endothelial growth factor
VSMCs	Vascular Smooth Muscle Cells

## References

[1] Wang X., Huang X. (2024). Risk factors and predictive indicators of rupture in cerebral aneurysms. Front. Physiol..

[2] Wu A., Zhao C., Mou S., Li S., Cui X., Zhang R. (2022). Integrated analysis identifies the IL6/JAK/STAT signaling pathway and the estrogen response pathway associated with the pathogenesis of intracranial aneurysms. Front. Immunol..

[3] Morel S., Schilling S., Diagbouga M.R., Delucchi M., Bochaton-Piallat M.L., Lemeille S., Hirsch S., Kwak B.R. (2021). Effects of Low and High Aneurysmal Wall Shear Stress on Endothelial Cell Behavior: Differences and Similarities. Front. Physiol..

[4] Nádasy G.L., Balla A., Dörnyei G., Hunyady L., Szekeres M. (2024). Direct Vascular Effects of Angiotensin II (A Systematic Short Review). Int. J. Mol. Sci..

[5] Dikalov S.I., Nazarewicz R.R. (2013). Angiotensin II-induced production of mitochondrial reactive oxygen species: Potential mechanisms and relevance for cardiovascular disease. Antioxid. Redox Signal..

[6] Edlinger C., Lichtenauer M., Wernly B., Pistulli R., Paar V., Prodinger C., Krizanic F., Thieme M., Kammler J., Jung C. (2019). Disease-specific characteristics of vascular cell adhesion molecule-1 levels in patients with peripheral artery disease. Heart Vessels.

[7] Gourdin M., Dubois P., Mullier F., Chatelain B., Dogné J.M., Marchandise B., Jamart J., De Kock M. (2012). The effect of clonidine, an alpha-2 adrenergic receptor agonist, on inflammatory response and postischemic endothelium function during early reperfusion in healthy volunteers. J. Cardiovasc. Pharmacol..

[8] Fan D., Fan T.J. (2017). Clonidine Induces Apoptosis of Human Corneal Epithelial Cells through Death Receptors-Mediated, Mitochondria-Dependent Signaling Pathway. Toxicol. Sci..

[9] Kataoka H. (2015). Molecular mechanisms of the formation and progression of intracranial aneurysms. Neurol. Med. Chir..

[10] Mosmann T. (1983). Rapid colorimetric assay for cellular growth and survival: Application to proliferation and cytotoxicity assays. J. Immunol. Methods.

[11] Miranda K.M., Espey M.G., Wink D.A. (2001). A rapid, simple spectrophotometric method for simultaneous detection of nitrate and nitrite. Nitric Oxide.

[12] Förstermann U., Sessa W.C. (2012). Nitric oxide synthases: Regulation and function. Eur. Heart J..

[13] Köksal Karayildirim Ç., Nalbantsoy A., Karabay Yavaşoğlu N.Ü. (2021). Prunetin inhibits nitric oxide activity and induces apoptosis in urinary bladder cancer cells via CASP3 and TNF-α genes. Mol. Biol. Rep..

[14] Dandona P., Dhindsa S., Ghanim H., Chaudhuri A. (2007). Angiotensin II and inflammation: The effect of angiotensin-converting enzyme inhibition and angiotensin II receptor blockade. J. Hum. Hypertens..

[15] Benicky J., Sánchez-Lemus E., Pavel J., Saavedra J.M. (2009). Anti-inflammatory effects of angiotensin receptor blockers in the brain and the periphery. Cell Mol. Neurobiol..

[16] Samuel N., Radovanovic I. (2019). Genetic basis of intracranial aneurysm formation and rupture: Clinical implications in the postgenomic era. Neurosurg. Focus.

[17] Giovannitti J.A., Thoms S.M., Crawford J.J. (2015). Alpha-2 adrenergic receptor agonists: A review of current clinical applications. Anesth. Prog..

[18] Atkinson G., Bianco R., Di Gregoli K., Johnson J.L. (2023). The contribution of matrix metalloproteinases and their inhibitors to the development, progression, and rupture of abdominal aortic aneurysms. Front. Cardiovasc. Med..

[19] Golombek S., Doll I., Kaufmann L., Lescan M., Schlensak C., Avci-Adali M. (2024). A Novel Strategy for the Treatment of Aneurysms: Inhibition of MMP-9 Activity through the Delivery of TIMP-1 Encoding Synthetic mRNA into Arteries. Int. J. Mol. Sci..

[20] Pei W., Zou Y., Wang W., Wei L., Zhao Y., Li L. (2018). Tizanidine exerts anti-nociceptive effects in spared nerve injury model of neuropathic pain through inhibition of TLR4/NF-κB pathway. Int. J. Mol. Med..

[21] Ma J., Li Y., Yang X., Liu K., Zhang X., Zuo X., Ye R., Wang Z., Shi R., Meng Q. (2023). Signaling pathways in vascular function and hypertension: Molecular mechanisms and therapeutic interventions. Signal Transduct. Target. Ther..

[22] Suarez-Roca H., Mamoun N., Sigurdson M.I., Maixner W. (2021). Baroreceptor Modulation of the Cardiovascular System, Pain, Consciousness, and Cognition. Compr. Physiol..

[23] Liu D., Hallt E., Platz A., Humblet A., Lassig-Smith M., Stuart J., Fourie C., Livermore A., McConnochie B.Y., Starr T. (2024). Low-dose clonidine infusion to improve sleep in postoperative patients in the high-dependency unit. A randomised placebo-controlled single-centre trial. Intensive Care Med..

[24] Nguyen A., Patel A.B., Kioutchoukova I.P., Diaz M.J., Lucke-Wold B. (2023). Mechanisms of Mitochondrial Oxidative Stress in Brain Injury: From Pathophysiology to Therapeutics. Oxygen.

[25] Chalouhi N., Ali M.S., Jabbour P.M., Tjoumakaris S.I., Gonzalez L.F., Rosenwasser R.H., Koch W.J., Dumont A.S. (2012). Biology of intracranial aneurysms: Role of inflammation. J. Cereb. Blood Flow Metab..

[26] Fukuda M., Aoki T. (2015). Molecular basis for intracranial aneurysm formation. Acta Neurochir. Suppl..

[27] Aoki T., Nishimura M., Matsuoka T., Yamamoto K., Furuyashiki T., Kataoka H., Kitaoka S., Ishibashi R., Ishibazawa A., Miyamoto S. (2011). PGE(2) -EP(2) signalling in endothelium is activated by haemodynamic stress and induces cerebral aneurysm through an amplifying loop via NF-κB. Br. J. Pharmacol..

[28] Fennell V.S., Kalani M.Y., Atwal G., Martirosyan N.L., Spetzler R.F. (2016). Biology of saccular cerebral aneurysms: A review of current understanding and future directions. Front. Surg..

[29] Chalouhi N., Points L., Pierce G.L., Ballas Z., Jabbour P., Hasan D. (2013). Localized increase of chemokines in the lumen of human cerebral aneurysms. Stroke.

[30] Flanders C.A., Rocke A.S., Edwardson S.A., Baillie J.K., Walsh T.S. (2019). The effect of dexmedetomidine and clonidine on the inflammatory response in critical illness: A systematic review of animal and human studies. Crit. Care.

[31] Taguchi K., Bessho N., Hasegawa M., Narimatsu H., Matsumoto T., Kobayashi T. (2018). Co-treatment with clonidine and a GRK2 inhibitor prevented rebound hypertension and endothelial dysfunction after withdrawal in diabetes. Hypertens. Res..

[32] Ferder L., Inserra F., Martínez-Maldonado M. (2006). Inflammation and the metabolic syndrome: Role of angiotensin II and oxidative stress. Curr. Hypertens. Rep..

[33] Brasier A.R., Recinos A., Eledrisi M.S. (2002). Vascular inflammation and the renin-angiotensin system. Arterioscler. Thromb. Vasc. Biol..

[34] Dechend R., Fiebler A., Lindschau C., Bischoff H., Müller D., Park J.K., Dietz R., Haller H., Luft F.C. (2001). Modulating angiotensin II-induced inflammation by HMG Co-A reductase inhibition. Am. J. Hypertens..

[35] Laaksamo E., Tulamo R., Liiman A., Baumann M., Friedlander R.M., Hernesniemi J., Kangasniemi M., Niemelä M., Laakso A., Frösen J. (2013). Oxidative stress is associated with cell death, wall degradation, and increased risk of rupture of the intracranial aneurysm wall. Neurosurgery.

[36] Starke R.M., Chalouhi N., Ali M.S., Jabbour P.M., Tjoumakaris S.I., Gonzalez L.F., Rosenwasser R.H., Koch W.J., Dumont A.S. (2013). The role of oxidative stress in cerebral aneurysm formation and rupture. Curr. Neurovasc. Res..

[37] Starke R.M., Raper D.M., Ding D., Chalouhi N., Owens G.K., Hasan D.M., Medel R., Dumont A.S. (2014). Tumor necrosis factor-α modulates cerebral aneurysm formation and rupture. Transl. Stroke Res..

[38] Shi Y., Li S., Song Y., Liu P., Yang Z., Liu Y., Quan K., Yu G., Fan Z., Zhu W. (2019). Nrf-2 signaling inhibits intracranial aneurysm formation and progression by modulating vascular smooth muscle cell phenotype and function. J. Neuroinflamm..

[39] Zhang J., Duan P., Nie B., Zhang Z., Shi R., Liu Q., Wang S., Xu T., Tian J. (2024). Identification and Verification of the Oxidative Stress-Related Signature Markers for Intracranial Aneurysm-Applied Bioinformatics. Front. Biosci. Landmark Ed..

